# Assessment of growth monitoring among children younger than 5 years at early childhood development centres in Nelson Mandela Bay, South Africa

**DOI:** 10.1002/hcs2.83

**Published:** 2024-02-05

**Authors:** Shawn W. McLaren, Liana Steenkamp, Jessica Ronaasen

**Affiliations:** ^1^ School of Human Sciences London Metropolitan University London UK; ^2^ Department of Dietetics Nelson Mandela University Gqeberha South Africa; ^3^ The Do More Foundation Westville South Africa

**Keywords:** early childhood development, CHWs, growth monitoring, wasting

## Abstract

**Introduction:**

Early childhood development (ECD) centres are important community hubs in South Africa and act as sites for community detection of childhood nutrition problems. This study aimed to assess the ability of trained ECD practitioners with optimal support to correctly classify the nutritional status of infants and young children at ECD centres in the Nelson Mandela Bay.

**Methods:**

A descriptive, cross‐sectional study was used to collect data from 1645 infants and children at 88 ECD centres. Anthropometric measurements were taken by trained fieldworkers and growth monitoring and promotion infrastructure was audited at ECD centres.

**Results:**

Of the sample, 4.4% (*n* = 72) were underweight by weight for age Z‐score (WAZ < −2) and 0.8% (*n* = 13) were severely underweight (WAZ < −3). Results showed that 13.1% (*n* = 214) were stunted by height for age Z‐score (HAZ < −2) and 4.5% (*n* = 74) were severely stunted (HAZ < −3). The prevalence of moderate acute malnutrition was 1.2% and severe acute malnutrition was 0.5%, while the prevalence of overweight was 9.2% and the prevalence of obesity was 4%. A significant level of agreement between the correct interpretation and the ECD practitioners' interpretation was observed across all the anthropometric indicators investigated. The true positive wasting cases had a mean mid‐upper arm circumference (MUAC) of 14.6 cm, which may explain the high false negative rate found in terms of children identified with wasting, where ECD practitioners fail to use the weight for height Z‐score (WHZ) interpretation for screening.

**Conclusion:**

By using ECD centres as hub to screen for malnutrition, it may contribute to the early identification of failure to thrive among young children. Although it was concerning that trained ECD practitioners are missing some children with an unacceptably high false negative rate, it may have been due to the fact that wasting in older children cannot be identified with MUAC alone and that accurate WFH plotting is needed. Onsite mentorship by governmental health workers may provide ECD practitioners with more confidence to screen children for growth failure based on regular WFH measurements. Moreover, ECD practitioners will be more confident to monitor the Road to Health booklets for missed vaccinations, vitamin A and deworming opportunities.

## Graphical abstract

This study aimed to assess the ability of trained early childhood development (ECD) practitioners with optimal support to correctly classify the nutritional status of infants and young children at ECD centres in the Nelson Mandela Bay. A significant level of agreement between the correct interpretation and the ECD practitioners' interpretation was observed across all the anthropometric indicators investigated.

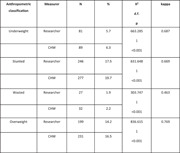

**Abbreviations**
CHW: community health worker
DSD: Department of Social Development
ECD: early childhood development
FN: false negative
FNR: false negative rate
FP: false positive
FPR: false positive rate
GMP: growth monitoring and promotion
HAZ: height for age Z‐score
IMCI: integrated management of childhood illness
MAM: moderate acute malnutrition
MUAC: mid‐upper arm circumference
NGO: nongovernmental organisation
RtHB: road to health booklet
SAM: severe acute malnutrition
SD: standard deviation
SPSS: statistical package for the social sciences
TN: true negative
TP: true positive
WAZ: weight for age Z‐score
WFH: weight for height
WHO: World Health Organisation
WHZ: weight for height Z‐score

## 1 INTRODUCTION

Childhood malnutrition continues to be a public health threat in sub‐Saharan Africa, where approximately 27.4% of children younger than 5 years are stunted, 25% are underweight and 10% are wasted [1]. Although the prevalence of stunting in southern Africa decreased from 31.8% in 1990 to 23.3% in 2020, there are still an estimated 1.6 million stunted children in this region. Apart from underweight, wasting and stunting, childhood overweight and obesity, associated with chronic diseases of lifestyle later in life, increased to 13.5% among schoolchildren aged 6–14 years, and should be seen as a public health concern [2]. Any form of malnutrition, especially stunting, can have a substantial negative impact on a child's cognitive development before the age of five, depriving them of the ability to reach their full potential and thrive in school [3].

Growth monitoring and promotion (GMP) is a nutrition surveillance strategy which is carried out in communities for early detection of childhood growth faltering [4]. Malnourished children are usually identified in the community by trained community health workers (CHWs), used in developing countries to address the shortage of skilled health workers [5]. The full assessment should include mid‐upperarm circumference (MUAC) measurement, plotting and interpreting weight for height, an examination of oedema, a clinical examination for Integrated Management of Childhood Illnesses (IMCI) danger signs and an appetite test [6]. However, CHWs lack adequate training, require better supervision and essential resources, and are sometimes inhibited from performing stabilisation care before referral due to restrictive policies [7]. As a result, in most areas in South Africa, screening by CHWs only include measurement and interpretation of MUAC and to assess for the presence of oedema, followed by referral to the nearest primary health care centre.

The space where children are cared for, often while their parents are at work or looking for work, are in early childhood development (ECD) centres, sometimes referred to as creches/preschools. In South Africa, approximately 1.6 million children are enroled in more than 42,000 ECD programmes [8]. It therefore makes sense for CHWs to include these centres in the outreach work to screen for malnutrition. Eliminating all malnutrition in children is an ambitious goal and should be a priority to multiple stakeholders including those working in education, health‐ and social development fields [9].

This study aimed to assess the ability of a nutrition‐trained ECD practitioner with optimal support to correctly classify the nutritional status of infants and young children at ECD centres in the Nelson Mandela Bay. A sub‐objective was to assess the growth monitoring practices performed at the ECD centres in the sample.

## 2 METHODS

### 2.1 Study design

**Figure 1:**
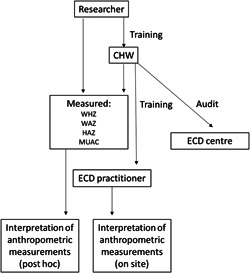
Study design. CHW, community health worker; ECD, early childhood development; WHZ, weight for height Z‐score; WAZ, weight for age Z‐score; HAZ, height for age Z‐score; MUAC, mid‐upper arm circumference.

This study used a descriptive, cross‐sectional design. Data were collected from 93 ECD centres in Motherwell, Nelson Mandela Bay between May 2017 and May 2018. ECD practitioners received nutrition mentorship from an NGO nutrition‐trained CHW (Figure 1). The CHW conducted site visits over a 9‐month period which started in May 2018 to screen children for malnutrition, as well as train, assess and support ECD practitioners with regard to growth monitoring at ECD centres. The average number of children per centre was 47.3, with an average of 3.5 teachers. This resulted in a teacher‐to‐child ratio of 1:13.5.

### 2.2 Data collection

CHWs were trained to take anthropometric measurements by an experienced, registered dietitian. Children were weighed by the field worker using a Seca electronic scale (Model 813) according to standardised procedures [6]. Weight was measured in kilograms (kg) to the nearest 0.1 kg. The fieldworkers also measured the height of children using a portable stadiometer (Seca model 213) using standardised procedures. Height was measured in centimetres (cm) to the nearest 0.1 cm. Mid‐upper arm circumference was measured according to standardised procedures [6], measured in centimetres (cm) to the nearest 0.1 cm.

Weight and height were physically plotted onto the road to health booklet (RtHB) clinic cards and growth charts on site by the ECD practitioner. Weight for age Z‐score (WAZ), height for age Z‐score (HAZ) and weight for height Z‐score (WHZ) were plotted.

Data were collected using a Google form on tablets. The forms were extracted to a Microsoft Excel spreadsheet and saved to a password protected Google Drive folder. Height, weight, MUAC, WAZ, HAZ and WHZ were captured using the form, in addition to the ECD practitioners' interpretation of the anthropometric measurements. The age of the children was calculated as the difference in months between the date of visit and child's date of birth as recorded from the clinic card. The gender of the child was captured to ensure use of the correct growth chart.

The field workers were trained to collect audit data from the ECD centres. This data included immunisation, deworming and vitamin A supplementation history from children's clinic card, as well as the availability of a height chart in the centre, whether the ECD practitioners were measuring and interpreting the height of children, whether ECD practitioners were able to measure and interpret MUAC and whether children were checked for oedema. ECD centre practitioners were interviewed about their referral to local clinic behaviour when malnutrition was detected.

### 2.3 Data analysis

The researchers then proceeded to recalculate the WAZ, HAZ and WHZ from the actual measurements using Anthro software [10]. These data were cleaned according to the World Health Organisation (WHO) [11] criteria. Participants with WAZ, HAZ or WHZ more than five standard deviations from the median were excluded from the analysis as these measurements are physiologically implausible and are more likely to be the result of measurement or data capture errors. Children with missing Z‐scores were removed from the data set.

WAZ, HAZ and WHZ were classified according to WHO criteria. For the purposes of comparison between the ECD practitioners' classification and measured anthropometric classification, WAZ < −2 was classified as underweight, HAZ < −2 was classified as stunted, WHZ < −2 was classified as wasted and WHZ > +2 was classified as overweight.

The ECD practitioners' interpretations of participant nutritional status were analysed and also used to generate classifications of underweight, stunted, wasted and overweight. Children described as ‘growing well’ were not classified as either underweight, stunted, wasted or overweight.

Data were analysed using SPSS v28 [12]. Data were described using means and standard deviations (SD) for continuous data, and frequencies and relative frequencies for categorical data. *χ* ^2^ tests were used to determine the level of agreement between the correct interpretation of anthropometry and ECD practitioner interpretations. Cohen's kappa statistic (*k*) was used to determine the strength of the agreement with significance set to *p* < 0.05. True positive (TP), false positive (FP), false negative (FN) and true negative (TN) values were calculated for the interpretations of weight for age, height for age and WHZ. The false positive rate (FPR) was calculated as FPR = FP/(FP+TN) and the false negative rate (FNR) was calculated as FNR = FN/(TP+FN). Sensitivity was calculated as sensitivity = TP/(TP+FN) and specificity was calculated as specificity = TN/(TN+FP). Figures were generated using RStudio [13].

### 2.4 Ethics

Ethical approval was obtained from the Nelson Mandela University Human Research Committee. Gatekeepers' permission was obtained from the Eastern Cape Department of Health. The study followed the ethical principles laid out in the Declaration of Helsinki [14]. Informed consent forms were signed by parents of participating children in advance of the study visit. Where cases of malnutrition were identified by fieldworkers, children were referred to the nearest clinic using a clinical referral form. Referral forms were given to the child's parents and follow‐up visits were conducted by fieldworkers.

## 3 RESULTS

The total sample included data for 2396 infants and young children. For the purpose of this paper, 690 participants were removed as they had missing data. A further 53 participants were removed as they had implausible Z‐scores. Finally, six records were removed as duplicate data. The final sample contained data for 1645 infants and young children.

The mean age (SD) of the participants was 42.05 months (12.06). The median age was 43.9 months. Approximately half of the sample was male (51.2%, *n* = 842). The mean (SD) WAZ −0.12 (1.2), the mean HAZ was −0.96 (1.26) and mean WHZ was 0.62 (1.28). Of the sample, 4.4% (*n* = 72) were underweight (WAZ < −2) and 0.8% (*n* = 13) were severely underweight (WAZ < −3). Results showed that 13.1% (*n* = 214) were stunted (HAZ < −2) and 4.5% (*n* = 74) were severely stunted (HAZ < −3). Moderate acute malnutrition (MAM) was prevalent among 1.2% (*n* = 19) of the children, while severe acute malnutrition (SAM) was prevalent among 0.5% (*n* = 8) of the participants. Of the total sample, 9.2% (*n* = 151) of the children were classified as overweight and 4.0% (*n* = 66) were in the obese category according to their WHZ.

A significant level of agreement between the correct interpretation and ECD practitioners interpretation was observed across all the anthropometric indicators investigated (Table 1). The level of agreement for wasting was lower than other indicators (*k* = 0.463), with the highest level of agreement observed for overweight (*k* = 0.769).

**Table 1:** Agreement between anthropometric interpretations of the researcher and early childhood development (ECD) practitioner.

Anthropometric classification	Measurer	*N* (male/female)	%	*X* ^2^ *d.f. p*	*k*
Underweight	Researcher	81 (40/41)	5.7	663.285	0.687
	ECD practitioner	89 (33/56)	6.3	1	
				<0.001	
Stunted	Researcher	246 (128/118)	17.5	631.648	0.669
	ECD practitioner	277 (126/151)	19.7	1	
				<0.001	
Wasted	Researcher	27 (14/13)	1.9	303.747	0.463
	ECD practitioner	32 (11/21)	2.2	1	
				<0.001	
Overweight	Researcher	199 (109/90)	14.2	836.615	0.769
	ECD practitioner	231 (127/104)	16.5	1	
				<0.001	

FP rates and FNRs are presented in Table 2. The FP rate for wasting was 1.3%, but the FNR for wasting was 52%. The FP rate is low across all anthropometric measurement interpretations, however, the FNR is high for underweight for age, stunting, wasting and overweight. The FNR is higher in younger children for underweight and stunting and appears to be more consistent across age groups for wasting and overweight.

**Table 2:** Accuracy of anthropometric interpretation by early childhood development (ECD) practitioners.

Indicator	Interpretation	Age (years)	True positive	False positive	False negative	True negative	False positive rate	False negative rate	Sensitivity	Specificity
Weight for age	Underweight	ALL	60	21	29	1292	0.016	0.32	67.42	98.40
		0–6	0	0	0	5	0	‐	‐	100.00
		6–12	1	0	1	15	0	0.5	50.00	100.00
		12–23	4	0	0	122	0	0	100.00	100.00
		24–35	16	11	5	295	0.036	0.238	76.19	96.41
		36–48	18	10	9	513	0.019	0.333	66.67	98.09
		>48	19	8	7	539	0.0146	0.269	73.08	98.54
Height for age	Stunted	ALL	191	86	55	1072	0.074	0.22	77.64	92.57
		0–6	1	0	1	3	0	0.5	50.00	100.00
		6–12	3	1	2	11	0.083	0.4	60.00	91.67
		12–23	16	4	13	93	0.041	0.448	55.17	95.88
		24–35	52	19	22	234	0.075	0.29	70.27	92.49
		36–48	55	28	31	436	0.06	0.36	63.95	93.97
		>48	59	34	20	460	0.06	0.25	74.68	93.12
Weight for height Z‐score	Wasted	ALL	13	18	14	1359	0.013	0.52	48.15	98.69
		0–6	0	0	0	5	0	‐	‐	100.00
		6–12	1	0	0	16	0	0	100.00	100.00
		12–23	0	0	1	125	0	1.0	0.00	100.00
		24–35	2	4	5	316	0.0125	0.714	28.57	98.75
		36–48	6	5	6	533	0.009	0.5	50.00	99.07
		>48	4	9	1	559	0.015	0.2	80.00	98.42
Weight for height Z‐score	Overweight	ALL	177	54	27	1144	0.045	0.13	86.76	95.49
		0–6	1	0	0	4	0	0	100.00	100.00
		6–12	5	0	1	11	0	0.166	83.33	100.00
		12–23	31	3	6	86	0.033	0.162	83.78	96.63
		24–35	30	15	7	275	0.052	0.189	81.08	94.83
		36–48	48	17	13	472	0.035	0.213	78.69	96.52
		>48	55	20	12	486	0.039	0.179	82.09	96.05

‐ indicates undefined.

**Figure 2:**
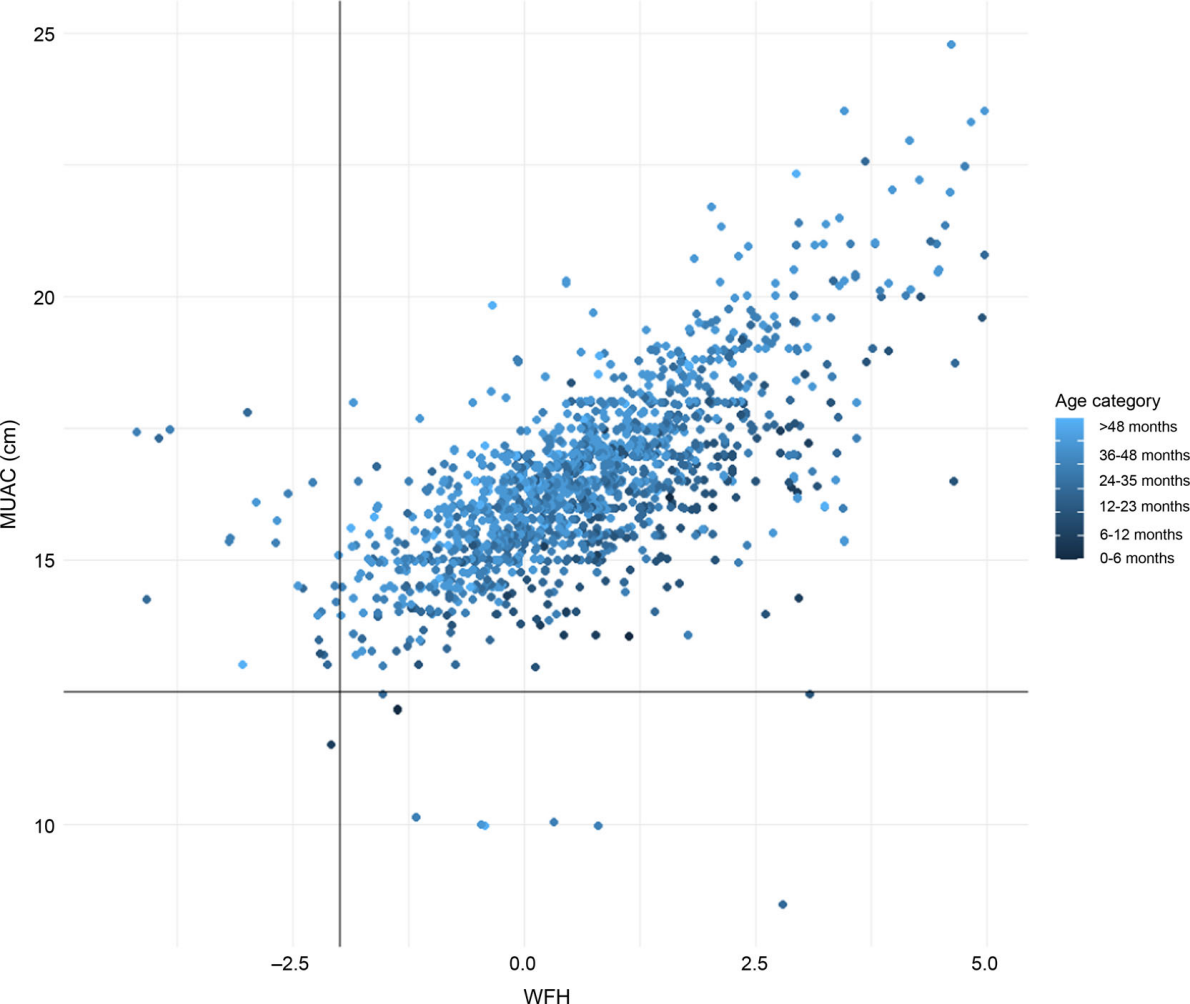
Weight for height (WFH) Z‐score plotted against mid‐upper arm circumference (MUAC) (cm) with cut‐off values (WHZ < −2, MUAC < 12.5 cm) for moderate acute malnutrition represented by vertical and horizontal lines.

Figure 2 displays MUAC (cm) plotted against WHZ for the children in the sample, disaggregated by age group. The mean MUAC among children identified as TP wasting cases by WHZ was 14.6 cm (*n* = 14), while the mean MUAC for FP cases was 15.35 cm (*n* = 11) and for FN cases was 15.51 cm (*n* = 9). The mean MUAC for TP cases of wasting identified by WHZ was 18.0 cm (*n* = 1573). More cases of MAM and SAM were identified by WHZ than MUAC, and only one child in this sample was positive for both indicators. An age bias for MUAC is apparent in the figure, with darker plots representing younger infants and children towards the lower part of the cluster, and lighter plots representing older children towards the top.

Three out of 88 ECD centres monitored the deworming and vitamin A supplementation of children, and clinic cards were available at 17 of 73 ECD centres, as shown in Table 3. Half of nutrition‐trained ECD practitioners were capable of carrying out a MUAC measurement. It was found that 54 of 88 ECD centres had a height chart available, but that height was measured by a trained ECD practitioner in only 17 of 88 ECD centres, as shown in Table 3.

**Table 3:** Road to health booklet (RtHB) monitoring at the nutrition‐trained early childhood development (ECD) centres. Unit: number of observations (count).

	RtHB available	Deworming checked	Vitamin A checked	Height chart available	Height measured by practitioner	Check for oedema	Practitioner able to do MUAC
No	73	85	85	35	67	68	45
Yes	17	3	3	54	17	22	45
Partially	0	2	2	1	1	0	0
Total	90	90	90	90	85	90	90

## 4 DISCUSSION

The results of this study suggest that trained ECD practitioners are capable of classifying the nutritional status of children at ECD centres in Nelson Mandela Bay when optimal support is available. Agreement between anthropometric interpretations of the researcher and ECD practitioner was statistically significant, however, there was a range in the level of agreement across the anthropometric indicators.

FPRs across all indicators of nutritional status were low, suggesting that few infants and young children will be inappropriately referred to a health centre for treatment. FPs are likely to be discharged back into the community on full assessment, incurring transport and opportunity costs for caregivers and undermining confidence in the health system [15]. However, there was a high FNR for WFH, while half of trained ECD centres were able to measure MUAC and a third of ECD centres were monitoring children for oedema. Therefore, the accuracy of all three acute malnutrition screening practices recommended by the WHO [6] is likely to be compromised in this setting. The MUAC values for children identified as wasted by WHZ were higher than the WHO cut‐off value for moderate wasting (12.5 cm). Therefore it is likely that these children would be missed by MUAC in this setting. While there is an inherent age‐bias in MUAC, in which infants and younger children have smaller arms and are therefore more likely to be identified as malnourished, the data from this sample suggests that even younger infants and children appear not acutely malnourished by MUAC. It may also be that older children with higher MUAC are more difficult for the ECD practitioners to screen and because their arms are thicker they do not look like children with low Z‐scores, making them more difficult to detect.

Despite the good level of agreement between researcher and ECD practitioner interpretation of HAZ, the FNR for stunting was 22%. Laar et al. [16] found that there was a moderate level of agreement in height measurements taken by CHWs and experienced measurers in Ghana. However, these researchers noted that errors were more likely to occur in measurements in crowded GMP sites and when health workers are overburdened [16]. Height charts were available in the majority of ECD centres included in the study, however, height was measured by a trained ECD practitioner in only 16 of the 84 centres. While the accuracy and frequency of height measurements of children in South African health settings is poor, a culture shift is required to address the double burden of malnutrition facing the country. South Africa has agreed to the Sustainable Development Goals, including goal 2.2, to end all forms of malnutrition, including stunting and overweight. While the country is on track to meet its targets for wasting, it is showing no progress or even a worsening situation with regard to stunting [17]. Currently, nutrition surveillance in South African secondary settings classifies children as SAM, MAM or not acutely malnourished, resulting in stunting and overweight lacking priority with the South African Department of Health. In addition, there is a lack of policy in place for treating stunting in children when it is identified. Fink and Rockers [18] have found that stunting during childhood is associated with diminished cognitive development and poorer schooling outcomes in later adolescence. Stunting has been associated with reduced human capital and intergenerational poverty [19]. While prevention of nutritional disorders and malnutrition and the associated developmental delays has been emphasised in the literature, researchers have also begun to suggest that the focus should shift from basic child survival towards thriving [19]. Therefore, Black et al. [20] have identified enabling environments as well as proximal components including safe and nutritious food, health care and learning opportunities as important parts of improving child survival, as well as ensuring that children grow and develop to meet their full potential [20].

In the current study, it was noted that only one ECD centre made referrals to the health service when a child was identified as malnourished. Reasons for the low rate of referrals by the ECD centres included ECD practitioners being too busy with educational activities, sporadic attendance at the ECD centre by children, and ECD practitioners preferring to wait for the CHW or nurses to complete the referral process. ECD practitioners also reported forgetting how to plot the growth charts as a barrier to making referrals. Similar limitations were noted by Blaauw et al. [21], where inadequate training, staff shortages and limited time were factors contributing to suboptimal use of the clinic cards among CHWs and healthcare workers. Based on the findings of Woldie et al. [22], CHWs can deliver high‐quality services, but their performance on complex tasks, such as diagnosis and counselling, tends to be actioned in practice at a lower standard and requires intentional support and training to achieve an adequate certain level of competency.

Biersteker et al. [23] identified that the National Department of Social Development (DSD), Basic Education and the Department of Health are the core governance pillars of the ECD sector. Collaboration is required between these departments to address the multidisciplinary nature of malnutrition. Closing the referral system link between the ECD centres which are now governed by the Department of Basic Education and the Department of Health primary health facility. It has been suggested that improved supervision of CHWs may improve their effectiveness [21]. Feroz et al. [24] have recommended the use of mobile phones to improve support and supervision for CHWs. However, these authors noted that problems with network connectivity and technical support can confound this approach [24]. It is important to consider that these forms of supervision and support may be less available to unregistered centres, who receive limited support from state authorities. Also in this study, the ECD practitioner had good support and mentorship which did not result in a better outcome in terms of reducing the FNRs in identifying wasting which warrants further investigation.

CHW have been referred to as a critical component of the South African health care system and are praised for their status within the community for their ability carry both indigenous knowledge and service community health issues at local level [25, 26]. ECD centres play a critical within local communities by providing parents and children with support regarding education and health and social protection [27]. ECD Practitioners may not be CWH, however, there are some similarities in the status they hold embedded within communities as teachers and caregivers trained in messages related to ECD.

The nutrition screening function described in this article could be fulfilled by community‐based individuals such as ECD practitioners and this supportive role enhances public health initiatives and can drive screening at a local community level [28]. A case study example of this occurring in practice is South African, Zero2Five Trusts' Mbizana stop stunting campaign 2020 using anthropometric measures of children at ECD centres to demonstrate how ECD practitioners, CHWs and local clinics can work together to improve child nutrition in their communities [29]. To appreciate the nature of this integrated discussion, one acknowledges the multidisciplinary nature of ECD and the ability for child health to fit comfortably under the umbrella of ECD. A shared responsibility for addressing the malnutrition crisis is a practical approach that is appropriate in developing countries where public health issues are complex and multidisciplinary in nature. Acknowledging the diverse scope of ECD and child well‐being as a fundamental element of the nurturing care framework necessary for young children that advocates for a collaborative approach, thereby creating partnership pathways for a practical and efficient strategy to tackle the malnutrition crisis in developing nations with complex public health issues.

## 5 CONCLUSION

This study aimed to assess the ability of trained ECD practitioners to correctly classify the nutritional status of infants and young children at ECD centres in the Nelson Mandela Bay and to quantify the nutrition screening practices at ECD centres in this community. The results of this study suggest that child health services can be promoted and encouraged by ECD centres, but require regular visits and support from local clinic staff. The link to local clinic staff requires active human resourcing and commitment to the community linkages by the Department of Health. Growth monitoring is difficult to implement in the absence of skilled support staff to assist with nutrition screening and referral. ECD centres are useful sites for community detection of malnutrition. In addition, overweight and obesity are prevalent in this community, but the opportunity for intervention is missed as this nutritional state is not captured or prioritised by the Department of Health. Onsite mentorship by the CHW proved valuable support for ensuring that key health messages were correctly understood and implement. However, an NGO cannot provide sustainable support and it is important that the Department of Health provide staff to become mentors to the ECD practitioners.

## AUTHOR CONTRIBUTIONS

**Shawn W. McLaren**: Formal analysis (lead); visualisation (lead); writing—original draft (lead); writing—review and editing (equal). **Liana Steenkamp**: Conceptualisation (lead); data curation (lead); formal analysis (supporting); funding acquisition (lead); investigation (lead); methodology (lead); project administration (lead); writing—review and editing (equal). **Jessica Ronaasen**: Formal analysis (supporting); investigation (equal); methodology (supporting); validation (supporting); writing—original draft (supporting); writing—review and editing (equal).

## CONFLICT OF INTEREST STATEMENT

The authors declare no conflict of interest.

## ETHICS STATEMENT

Ethical approval was obtained from Nelson Mandela University Human Research Ethics Committee.

## INFORMED CONSENT

Informed consent forms were signed by parents of participating children in advance of the study visit.

## Data Availability

Data are available on request from the authors.

## References

[1] UNICEF, WHO and World Bank . Joint child malnutrition estimates (online). 2022. [cited 2022 Nov 17]. Available from: https://data.unicef.org/resources/dataset/malnutrition-data/

[2] Shisana O , Labadarios D , Rehle T , Simbayi L , Zuma K , Dhansay A , et al. South African National Health and Nutrition Examination Survey (SANHANES‐1). Cape Town: HSRC Press; 2013.

[3] Ronaasen JE , Steenkamp L , Wilson TM , Venter D , Elkonin D . ECD indicators and nutritional status of grade R children: an interest to social workers. Southern Afr J Soc Work Soc Dev. 2018;29(2):1–13. 10.25159/2415-5829/981

[4] Ashworth A , Shrimpton R , Jamil K . Growth monitoring and promotion: review of evidence of impact. Matern Child Nutr. 2008;4(Suppl 1):86–117. 10.1111/j.1740-8709.2007.00125.x PMC6860476 18289158

[5] Lehmann U , Sanders D . Community health workers: what do we know about them? The state of the evidence on programmes, activities, costs and impact on health outcomes of using community health workers. Geneva: World Health Organisation; 2007.

[6] WHO . Guideline: updates on the management of severe acute malnutrition in infants and children. Geneva: World Health Organization; 2013. 24649519

[7] Mambulu‐Chikankheni FN , Eyles J , Ditlopo P . Exploring the roles and factors influencing community health workers' performance in managing and referring severe acute malnutrition cases in two subdistricts in South Africa. Health Soc Care Commun. 2018;26(6):839–848. 10.1111/hsc.12595 30047600

[8] Department of Basic Education . ECD Census 2021: Report. Pretoria: Department of Basic Education; 2022.

[9] Makanjana O , Naicker A . Nutritional status of children 24–60 months attending early child development centres in a semi‐rural community in South Africa. Int J Environ Res Public Health. 2020;18(1):261. 10.3390/ijerph18010261 33396403 PMC7795561

[10] World Health Organisation . Software for assessing growth and development of the world's children. 2006; Geneva: WHO. [cited 2017 Jun 27]. Available from: http://www.who.int/childgrowth/software/WHOantho2005_PC_Manual.pdf

[11] WHO . WHO multicentre growth reference study group: WHO child growth standards: length/height for age, weight for age, weight for length, weight for height and body mass index for age: methods and development. Geneva: World Health Organisation; 2006.

[12] IBM Corp. Released . IBM SPSS statistics for Windows. Version 28.0. Armonk, NY: IBM Corp; 2021.

[13] RStudio Team . RStudio: integrated development for R. Boston, MA: R Studio, PBC; 2023. http://www.rstudio.com/

[14] World Medical Association . World medical association declaration of helsinki: ethical principles for medical research involving human subjects. JAMA. 2013;310(20):2191–2194. 10.1001/jama.2013.281053 24141714

[15] Kapil U , Pandey RM , Bansal R , Pant B , Varshney AM , Yadav CP , et al. Mid upper arm circumference in detection of weight‐ for‐height Z‐score below ‐3 in children aged 6–59 months. Public Health Nutr. 2018;21(10):1794–1799. 10.1017/S1368980017004165 29397809 PMC10260960

[16] Laar ME , Marquis GS , Lartey A , Gray‐Donald K . Reliability of length measurements collected by community nurses and health volunteers in rural growth monitoring and promotion services. BMC Health Serv Res. 2018;18(1):118. 10.1186/s12913-018-2909-0 29454360 PMC5816548

[17] UNICEF, World Health Organisation, World Bank Group . Joint child malnutrition estimates, key findings of the 2021 edition. 2021. Available from: https://www.who.int/publications/i/item/9789240025257. Accessed 12 Oct 2023.

[18] Fink G , Rockers PC . Childhood growth, schooling, and cognitive development: further evidence from the young lives study. Am J Clin Nutr. 2014;100(1):182–188. 10.3945/ajcn.113.080960 24808488

[19] Prendergast AJ , Humphrey JH . The stunting syndrome in developing countries. Paediatr Int Child Health. 2014;34(4):250–265. 10.1179/2046905514Y.0000000158 25310000 PMC4232245

[20] Black MM , Lutter CK , Trude ACB . All children surviving and thriving: re‐envisioning UNICEF's conceptual framework of malnutrition. Lancet Global Health. 2020;8(6):e766–e767. 10.1016/S2214-109X(20)30122-4 32446344

[21] Blaauw R , Daniels L , Plessis LD , Koen N , Koornhof HE , Marais M , et al. Assessing the utilisation of a child health monitoring tool. S Afr N J Child Health. 2017;11:174–179. 10.7196/SAJCH.2017.V11I4.1326

[22] Woldie M , Feyissa GT , Admasu B , Hassen K , Mitchell K , Mayhew S , et al. Community health volunteers could help improve access to and use of essential health services by communities in LMICs: an umbrella review. Health Policy Plan. 2018;33(10):1128–1143. 10.1093/heapol/czy094 30590543 PMC6415721

[23] Biersteker L , Dawes A , Hendricks L , Tredoux C . Center‐based early childhood care and education program quality: a South African study. Early Child Res Q. 2016;36:334–344. 10.1016/j.ecresq.2016.01.004

[24] Feroz A , Jabeen R , Saleem S . Using mobile phones to improve community health workers performance in low‐and‐middle‐income countries. BMC Public Health. 2020;20(1):49. 10.1186/s12889-020-8173-3 31931773 PMC6958627

[25] Murphy JP , Moolla A , Kgowedi S , Mongwenyana C , Mngadi S , Ngcobo N , et al. Community health worker models in South Africa: a qualitative study on policy implementation of the 2018/19 revised framework. Health Policy Plan. 2021;36(4):384–396. 10.1093/heapol/czaa172 33367608 PMC8128020

[26] Thomas LS , Buch E , Pillay Y . An analysis of the services provided by community health workers within an urban district in South Africa: a key contribution towards universal access to care. Hum Resour Health. 2021;19(1):22. 10.1186/s12960-021-00565-4 33602255 PMC7889710

[27] Ebrahim H , Irvine M . The South African National Curriculum Framework for children from birth to four. 2018. Available from: https://www.education.gov.za/Portals/0/Documents/curriculum%20docs/NCF%202018/NCF%20English%202018%20web.pdf?ver=2018-05-14-124718-317

[28] Laurenzi CA , Skeen S , Coetzee BJ , Notholi V , Gordon S , Chademana E , et al. Instructive roles and supportive relationships: client perspectives of their engagement with community health workers in a rural South African home visiting program. Int J Equity Health. 2021;20(1):32. 10.1186/s12939-020-01377-z 33436011 PMC7805205

[29] Thorogood G. Food and nutrition security for the preschool child: enhancing early childhood development. In: May J , Witten C , Lake L , editors. South African Child Gauge 2020. Cape Town: Children's Institute, University of Cape Town; 2022. p. 96–110.

